# Pyorrhœa Alveolaris: Its Causes and Treatment

**Published:** 1903-04

**Authors:** Eugene J. Wetzel

**Affiliations:** D.M.D. (Harvard), Mulhouse, Germany


### PYORRHŒA ALVEOLARIS: ITS CAUSES AND TREATMENT.[1]

BY EUGENE J. WETZEL, D.M.D. (HARVARD), MULHOUSE, GERMANY.

Pyorrhoea alveolaris, like most other well-defined diseases, is not a constitutional disease, but a local one, and requires local treatment. It appears in two distinct forms. First, the disease commences at the apex of the roots of the teeth without pus formation. The second form commences at the gingival border or margin around the necks of the teeth, with considerable pus formation. Both kinds lead to the entire loss of the teeth if not treated, and ought to be checked at once by the dentist if there are traces to be found.

Physiologists and pathologists are still investigating to find the cause, but I think no man better than the dentist can discover its origin.

The first form is caused by the lack of work and pressure on the teeth. The second is caused by the neglect of cleanliness of the oral cavity. Both kinds can be cured if not too far advanced, and will remain so if instructions as to care, cleanliness, free use of tooth-brush, etc., are carefully observed by the patient. The first step in the treatment of the first kind is to test the strength of the teeth in their sockets, and if any tooth is too loose it should be removed. Then clean the oral cavity thoroughly with a three per cent. solution of hydrogen dioxide or with a 2000 solution of sublimate. Then instruct your patient to exercise some pressure on the teeth and the jaws by masticating the food thoroughly. All soft food should be avoided, and explain to the patient that the teeth are the hardest substance of our bodies, and are therefore there as instruments to do hard work, and not as ornaments only. The best

---

[1] Read at the thirty-first annual meeting of the Harvard Dental Alumni Association, June 23, 1902.

work and food for the teeth is old stale bread without any butter or any lubricant on it. This cleans the teeth better than any brush. Between meals the patient can chew occasionally some licorice-wood. The patient must be watched and encouraged to persevere. At the beginning of the treatment it is advisable to see your patient twice a week. At each sitting the gums have to be frictioned with hydrogen dioxide, and then make use of tincture of iodine, two-thirds, and zinc chloride, one-third, to stimulate. If those instructions are minutely executed there is generally a great improvement of the teeth after from four to six weeks.

The second form, with pus formation and pockets from beneath the free edges of the gums, is more frequently found. It is generally caused by the neglect of cleanliness, by local irritants such as calculus, necrosis of the alveolar process border. This kind of pyorrhœa alveolaris can be better cured than the one mentioned just now. The treatment is a more serious operation, which consists in the thorough removal of all local irritants such as tartar. This operation is generally not accomplished in one sitting, for at each successive sitting you have to probe carefully again and again to make sure of completeness. When this has been done then make free use of hydrogen dioxide three per cent. solution, or a 2000 solution of sublimate to wash thoroughly out the pockets of the gum. Then discharge your patient and recommend the brushing of the gums and teeth well twice a day. After the first sitting it is wise not to make an appointment for the next day, but for four or five days later, so that the healing process can take place. It is marvellous to see sometimes the change that is produced only after the first sitting, and without exaggeration there are cases where pyorrhœa has completely disappeared. Teeth that are pretty loose ought to be tied together; the new formation has better chance to take place. Into the pockets I introduce, with a fine nerve instrument and very little cotton wrapped around the point, some tincture of iodine, two-thirds, and zinc chloride, one-third, and continue so at each sitting. Formalin might be used if the suppuration does not stop with the first tincture.

We often have cases where the first or second upper molar is badly loosened on the palatine root; the buccal roots are quite healthy. In such cases I advise the complete removal of the palatine root, an operation which is easily done with the engine and a fissure bur. (Querhibe.)

After a complete cure has been established the patient must be advised to be examined once a month and have a treatment of the gums made with tincture of iodine and chloride of zinc.

### HOW TO CONTROL THE ACIDITY IN THE MOUTH.

We have a great many patients from the age of fourteen years upward who suffer terribly from the ravages of caries. Most dentists cure the disease by filling all decayed teeth, but neglect to make the patients attentive to the cause, which generally is in the saliva. When I began to practise, thirteen years ago, I advised my patients to wash out their mouths very often with the solution of bicarbonate of soda, and the results were pretty good. But wishing still a better success, I have some very hard lozenges (tabloids) of bicarbonate of soda made, and advise my patients to put one quarter of a lozenge (or tabloid) between the upper molar and the cheek before going to bed, changing alternately, one evening on the right, another evening on the left side of the mouth. In this way the bicarbonate of soda dissolves very slowly all night and neutralizes the acid saliva.

